# Supplementary material for: Content-rich biological network constructed by mining PubMed abstracts
Source: BMC Bioinformatics. 2004 Oct 8;5:147. doi: 10.1186/1471-2105-5-147 (PMC528731; doi:10.1186/1471-2105-5-147)
Supplement: Additional File 2 — The original results of the above study (non-essential files are deleted to keep the file size under the limit set by BMC bioinformatics). [file 1471-2105-5-147-S2.bz2 › chilibotAdditionalFile2/dip50/448ID9115216E10455/html/CCR1_CCL2.html]

 


 **CCR1** and **CCL2** 
  
Found 155 abstracts in PubMed, retrieved 50.  
 

 What does Google say? 
 PDF only 
| .edu only 

---

**Interactive relationship** (e.g. stimulation, inhibition, etc)

**Neutral relationship**- Functional studies and binding experiments using biotinylated monocyte chemoattractant protein MCP 1  [ **CCL2** ]   **CCL2**  indicate that  **CCL2**  could be a candidate chemokine ligand for glial L CCR  [ **CCR1** ] .  Ref: 12555200 Glia, 2003
- Expression of L CCR  [ **CCR1** ]  in HEK 293 cells reveals functional responses to  **CCL2** , CCL5, CCL7, and CCL8.  Ref: 12885941 J Leukoc Biol, 2003
- The biological effects of monocyte chemoattractant protein MCP 1  [ **CCL2** ]  are mediated by binding to C C chemokine receptor CCR  [ **CCR1** ]  2.  Ref: 11981759 Hepatology, 2002
- Biotinylated  **CCL2**  binds to L CCR  [ **CCR1** ]  expressing cells, and transfection experiments with an L CCR  [ **CCR1** ]  green fluorescent protein fusion protein showed L CCR  [ **CCR1** ]  expression in the membranes of recombinant HEK 293 cells.  Ref: 12885941 J Leukoc Biol, 2003
- The G protein coupled receptor CCR  [ **CCR1** ]  2B is probably the most important MCP 1  [ **CCL2** ]  receptor in vivo, and loss of MCP 1  [ **CCL2** ]  effector function alone is sufficient to impair monocytic trafficking in inflammation models.  Ref: 12556201 Expert Opin Ther Targets, 2003
- Monocyte chemoattractant protein 1  [ **CCL2** ]  MCP 1  [ **CCL2** ] , acting through its C C chemokine receptor 2 CCR  [ **CCR1** ]  2, has important roles in inflammation, angiogenesis, and wound repair.  Ref: 11641460 Pediatr Res, 2001
- We conclude that although hyperoxia increases MCP 1  [ **CCL2** ]  in young piglet lungs, it also decreases CCR  [ **CCR1** ]  2 abundance, which may limit participation of MCP 1  [ **CCL2** ]  in alveolar macrophage recruitment.  Ref: 11641460 Pediatr Res, 2001
- The present findings suggest that MCP 1  [ **CCL2** ] , RANTES, and IP 10 may participate in the pathogenesis of mercuric chloride induced tubulointerstitial fibrosis with mononuclear cell infiltration, via CCR2,  **CCR1**  or CCR5, and CXCR3, respectively.  Ref: 12834626 Exp Mol Pathol, 2003

**Non-interactive relationship** (e.g. studied together, co-existance, homology, etc.)

- Only CpG ODN exacerbated HAF GN with an increase of glomerular macrophages, which was associated with massive albuminuria and increased renal MCP 1  [ **CCL2** ]   **CCL2** , RANTES CCL5,  **CCR1** , CCR2, and CCR5 mRNA expression.  Ref: 12538732 J Am Soc Nephrol, 2003
- Chemokine inducible transcripts could be clustered in functional groups, including selected cytokines and receptors e.g., IL 1beta,  **CCL2**  monocyte chemotactic protein 1  [ **CCL2** ] , and the CCL5 receptor  **CCR1**  and molecules involved in extracellular matrix recognition and digestion e.g., CD44 splice transcripts, urokinase type plasminogen activator receptor, matrix metalloprotease MMP 9, and MMP 19.  Ref: 11907119 J Immunol, 2002
- The mRNAs for MIP 1alpha and MCP 1  [ **CCL2** ] , as well as their cognate receptors  **CCR1**  and CCR2, respectively, were localized in resident and infiltrating macrophages in numbers commensurate with those of F4 80 immunopositive macrophages in adjacent tissue sections.  Ref: 12444600 J Neurosci Res, 2002
- Although certain transcripts were stimulated by both agonists e.g., IL 1beta and  **CCL2** , others were induced only by either LPS e.g., TNF alpha and IL 6 or CCL5 e.g., MMP 19 or were divergently regulated e.g.,  **CCR1** .  Ref: 11907119 J Immunol, 2002
- CD68 expression correlated with MIP 1alpha R  [ **CCR1** ] =0.39, P=0.01, but not with MCP 1  [ **CCL2** ]  expression.  Ref: 11561108 Rheumatology (Oxford), 2001
- We used radioligand binding and 35S GTPgammaS exchange assays in membranes from a cell line transfected to express  **CCR1**  Ba F3 hCCR1 to characterize a panel of chemokines HCC 1, MIP 1alpha, MIP 1beta, MIP 1delta, MPIF 1, MCP  [ **CCL2** ]  2, MCP  [ **CCL2** ]  3, and RANTES as  **CCR1**  ligands.  Ref: 12381680 Br J Pharmacol, 2002
- CD14 DCs retain unique features of tissue DCs, as evidenced by the gene expression profile of no CCR7 but more  **CCR1**  and no TARC but abundant MCP1  [ **CCL2** ]  and Eta 1.  Ref: 12176896 Blood, 2002
- In a prospective study using flow cytometry, we examined percentages of blood mononuclear cells MNC expressing the chemokines RANTES, MCP 1  [ **CCL2** ] , MIP 1alpha, MIP 1beta, and IL 8, the chemokine receptor CCR  [ **CCR1** ]  5, the T cell activation marker CD25, and the T cell chemoattractant IL 16 in patients with WAD and, for reference, in healthy controls.  Ref: 11506197 J Clin Immunol, 2001
- The mRNA expression of CC chemokines predominantly monocyte chemoattractant protein 1  [ **CCL2** ]  MCP 1  [ **CCL2** ]   **CCL2** , RANTES CCL5 and their receptors  **CCR1** , CCR2, CCR5 increased progressively from days 2 to 10.  Ref: 11373340 J Am Soc Nephrol, 2001
- Because MCP 1  [ **CCL2** ]  gene is up regulated by oxidants, we hypothesized that inhaled NO with hyperoxia will increase MCP 1  [ **CCL2** ]  production and CCR  [ **CCR1** ]  2 expression more than either gas alone.  Ref: 11641460 Pediatr Res, 2001
- In parallel,  **CCR1**  and CCR5 expression is down regulated and CXCL8,  **CCL2** , CCL3 and CCL5 production is up regulated.  Ref: 12548563 Eur J Immunol, 2003
- In this study, we report that receptor phosphorylation also provides a stimulatory signal for CCR  [ **CCR1** ]  ligand 2  **CCL2**  production.  Ref: 11564766 J Immunol, 2001
- We studied polymorphisms in the genes for monocyte chemoattractant protein 1  [ **CCL2** ]  MCP 1  [ **CCL2** ]  and CC chemokine receptor CCR  [ **CCR1** ]  2 in 171 Parkinson s disease PD patients and 340 controls.  Ref: 12889089 Mov Disord, 2003
- In J774 cells, CpG ODN induced the chemokines MCP 1  [ **CCL2** ]   **CCL2**  and RANTES CCL5 and the chemokine receptors  **CCR1**  and CCR5.  Ref: 12538732 J Am Soc Nephrol, 2003
- In the tumor stroma, the major source of MCP 1  [ **CCL2** ] , TP and CCR  [ **CCR1** ]  2 was macrophages, and the sites of positive staining for MCP 1  [ **CCL2** ] , TP and CCR  [ **CCR1** ]  2 matched in most of the myxomas.  Ref: 12520153 Circ J, 2003
- METHODS Expression of the 4 C C chemokines monocyte chemoattractant protein 1  [ **CCL2** ]  MCP 1  [ **CCL2** ] , macrophage inflammatory protein 1alpha MIP 1alpha, MIP 1beta, and RANTES, and their receptors CCR  [ **CCR1** ]  2 and CCR  [ **CCR1** ]  5, was assessed in 11 OA patients and 5 normal controls, by reverse transcriptase polymerase chain reaction RT PCR, enzyme linked immunosorbent assay ELISA, immunochemistry, and flow cytometry on untreated or interleukin 1beta IL 1beta and or tumor necrosis factor alpha TNFalpha stimulated chondrocytes.  Ref: 11352237 Arthritis Rheum, 2001
- The individual and combined effects of inhaled nitric oxide NO and hyperoxia on lung MCP 1  [ **CCL2** ]  and CCR  [ **CCR1** ]  2 in relation to lung leukocyte dynamics are unknown.  Ref: 11641460 Pediatr Res, 2001
- In EAE protected mice, 17 beta estradiol strongly inhibited mRNA expression of the chemokines RANTES, MIP 1 alpha, MIP 2, IP 10, and MCP 1  [ **CCL2** ] , and of the chemokine receptors  **CCR1** , CCR2 and CCR5 at both time points.  Ref: 11550221 J Neurosci Res, 2001
- At 26 weeks postirradiation, messages encoding the chemokines BLC now known as Scyb13, C10 now known as Scya6, IP 10 now known as Scyb10, MCP 1  [ **CCL2** ]  now known as Scya2  [ **CCL2** ] , MCP  [ **CCL2** ]  3 now known as Scya7, MIP 1gamma now known as Scya9, and RANTES now known as Scya5 and the chemokine receptors  **Ccr1** , Ccr2, Ccr5 and Ccr6 were elevated in fibrosis sensitive C57BL 6 mice.  Ref: 11839087 Radiat Res, 2002
- Monocyte chemoattractant protein 1  [ **CCL2** ]  and its receptor CCR  [ **CCR1** ]  2 in piglet lungs exposed to inhaled nitric oxide and hyperoxia.  Ref: 11641460 Pediatr Res, 2001
- Synthetic MCP  [ **CCL2** ]  analogs corresponding to the MMP cleaved forms bound CC chemokine receptor CCR  [ **CCR1** ]  2 and CCR  [ **CCR1** ]  3, but lacked chemoattractant activity in pre B cells transfected with CCR  [ **CCR1** ]  2 and CCR  [ **CCR1** ]  3 or in THP 1 monocytic cells, a transformed leukemic cell line.  Ref: 12149192 Blood, 2002
- Several selective small molecular weight CCR  [ **CCR1** ]  2B antagonists and MCP 1  [ **CCL2** ]  blocking antibodies have been described.  Ref: 12556201 Expert Opin Ther Targets, 2003
- Paraffin embedded specimens of 17 resected cardiac myxomas were immunohistochemically stained for MCP 1  [ **CCL2** ] , CC chemokine receptor 2 CCR  [ **CCR1** ]  2, TP, CD31, and CD68.  Ref: 12520153 Circ J, 2003
- Twenty four hours after induction of the GN, the glomerular mRNA expression of the chemokine monocyte chemoattractant protein 1  [ **CCL2** ]  MCP 1  [ **CCL2** ]  and the cognate chemokine receptor CCR  [ **CCR1** ]  2 were examined by Northern blotting and RT PCR.  Ref: 12110006 Kidney Int, 2002
